# CMS GUIDE Model: Lessons Learned By An Established Track Program At End Of Year One

**DOI:** 10.1093/geroni/igaf122.3561

**Published:** 2025-12-31

**Authors:** Jessica Mongelli, Kaitlin Tangredi, Marzena Gieniusz, Christian Nouryan, Christie Ulbricht, Megan Carroll, Maria Carney, Edith Burns

**Affiliations:** Northwell Health, New Hyde Park, New York, United States; Northwell Health, New Hyde Park, New York, New York, United States; Northwell Health, New Hyde Park, New York, United States; Northwell Health, Great Neck, New York, United States; Northwell Health, New Hyde Park, New York, United States; Northwell Health, New Hyde Park, New York, United States; Zucker School of Medicine at Hofstra/Northwell, New Hyde Park, New York, United States; Zucker School of Medicine Hofstra-Northwell, Manhasset, New York, United States

## Abstract

‘Guiding an Improved Dementia Care Experience’ (GUIDE) is a CMS Innovation Center pilot program implemented January 2024, as an alternate payment model for interprofessional teams to provide comprehensive dementia care for people living with dementia and their caregivers. Program criteria encompass screening and recruitment, comprehensive and home safety assessment, ongoing beneficiary monitoring, data collection/management/reporting, respite, caregiver supports, etc. We report first-year implementation and evolution as an ‘Established Track’ program in a large integrated health system in a major metropolitan area. To date, the program has enrolled 158 beneficiaries and their caregivers; 19% low, 73% moderate, 8% high complexity. Average age of beneficiaries 79 years, caregiver relationship 53% child, 35% spouse, 10% other. Over 35% of eligible beneficiaries have utilized respite services. Approximately 9% have disenrolled, the majority (64%) expired/hospice. An adaptable team-based approach has been a critical strategy for success. Helpful processes that have evolved include structured recurring meetings, weekly review of data and submissions, care coordination and scheduled monitoring of required care-delivery services. Education and outreach with all stakeholders (e.g. interprofessional healthcare providers, lay community) is essential. Internal challenges have included need for ongoing adjustment of workflows and processes, and system constraints on structuring database/submission/reporting. Additional challenges include changing program requirements, recognizing and addressing the need to supplement internal resources (e.g. home visits, respite care), and identifying and establishing partnerships with external entities. These successes and challenges may significantly impact effectiveness of this pilot model program. Providing additional resources for this vulnerable population requires a true interprofessional, multidisciplinary approach.

